# Proactive prophylaxis with azithromycin and hydroxychloroquine in hospitalized patients with COVID-19 (ProPAC-COVID): a statistical analysis plan

**DOI:** 10.1186/s13063-020-04795-0

**Published:** 2020-10-20

**Authors:** Pradeesh Sivapalan, Charlotte Suppli Ulrik, Therese Sophie Lappere, Josefin Viktoria Eklöf, Saher Burhan Shaker, Uffe Christian Steinholtz Bødtger, Andrea Browatzki, Christian Niels Meyer, Ulla Møller Weinreich, Christian B. Laursen, Tor Biering-Sørensen, Filip Krag Knop, Jens D. Lundgren, Jens-Ulrik Stæhr Jensen

**Affiliations:** 1 Herlev and Gentofte Hospital, University of Copenhagen, Copenhagen, Denmark; 2 Amager and Hvidovre Hospital, University of Copenhagen, Copenhagen, Denmark; 3 Bispebjerg and Frederiksberg Hospital, University of Copenhagen, Copenhagen, Denmark; 4 Næstved, Slagelse and Ringsted Hospitals, University of Southern Denmark, Odense, Denmark; 5 Nordsjællands Hospital Hillerød, University of Copenhagen, Hillerød, Denmark; 6 Zeeland University Hospital, Roskilde, Denmark; 7 Aalborg University Hospital, Aalborg University, Aalborg, Denmark; 8 Odense University Hospital, University of Southern Denmark, Odense, Denmark; 9 Rigshospitalet, University of Copenhagen, Copenhagen, Denmark

**Keywords:** Detailed statistical analysis plan, Randomized controlled trial, Infectious diseases, Safety, Hydroxychloroquine, Azithromycin, Intervention

## Abstract

**Background:**

There is an urgent need for treatments that can shorten hospitalization and lower the risk of secondary infection and death in patients with corona disease. The ProPac-COVID trial evaluates whether combination therapy with macrolide azithromycin and hydroxychloroquine via anti-inflammation/immune modulation, antiviral efficacy, and pre-emptive treatment of supra-infections can shorten hospitalization duration and reduce the risk of non-invasive ventilation, treatment in the intensive care unit, and death in patients with acute hospital admission and a positive test for 2019-nCoV and symptoms of COVID-19 disease.

**Methods:**

The ProPAC-COVID is a multi-center, randomized, placebo-controlled, double-blinded clinical trial. The primary outcome is number of days spent alive and out of hospital within 14 days from randomization. Randomization will be in blocks of unknown size, and the final allocation will be stratified for age, site of recruitment, and whether the patient has any chronic lung diseases. Data is analyzed using intention-to-treat (ITT) principles, and main analyses will also be subject to modified ITT analysis and per protocol analysis.

**Discussion:**

This paper describes the detailed statistical analysis plan for the evaluation of primary and secondary endpoints of the ProPAC-COVID study. Enrolment of patients to the ProPAC-COVID study is still ongoing. The purpose of this paper is to provide primary publication of study results to prevent selective reporting of outcomes, data-driven analysis, and to increase transparency.

**Trial registration:**

ClinicalTrials.gov NCT04322396. Registered on 26 March 2020.

## Background

In the ongoing coronavirus disease 2019 (COVID-19) pandemic that arose in Wuhan, China, there is still sparse knowledge of the course, risk of complications, and how hospitalized patients are best treated to ensure best possible survival and shortest duration of hospitalization. Presently, symptomatic and organ supportive therapy, including assisted ventilation in acute hypoxic respiratory failure, is recommended [1]. A high incidence of bacterial super-infections in patients with COVID-19 has been reported, and patients with more severe COVID-19 seem to have a high risk of death due to septic shock [2]. The length of hospitalization is observed to be relatively long, 10–15 days [3], which in itself is a problem as hospitals can quickly reach the maximum capacity for hospitalization and the proportion of patients who become critically ill have, on the observations reported so far, had a mortality rate of > 60% [4], and overall mortality for admitted patients in China with COVID-19 infection is apparently unusually high for viral respiratory tract infections, up to 25% [5].

Thus, there is an urgent need for treatments that can improve patients’ outcomes in COVID-19 including lower risk of secondary infection, death and shorter duration of hospital admission. The study will clarify whether drug treatment with azithromycin in combination with hydroxychloroquine for 15 days from hospitalization with diagnosed COVID-19 infection in hospitalized patients may reduce the length of hospitalization, the risk of hospitalization in the intensive care unit (ICU), treatment with non-invasive ventilation (NIV), and death. The study will also clarify whether this treatment can reduce the need for oxygen supplementation (time for breathing on its own without oxygen supplementation) or for regular long-term oxygen therapy oxygen supplementation (“home oxygen”).

### Research hypothesis

In patients with acute hospital admission, a positive test for SARS-CoV-2 and symptomatic COVID-19 and treatment with virus-modifying drug hydroxychloroquine as well as virus-immunomodulatory and antibacterial drug azithromycin can lead to shorter hospitalization and fewer admissions to the ICU.

### Study objectives

The objective of this randomized, placebo-controlled, double-blinded multi-center trial is to investigate whether 15-day treatment with azithromycin and hydroxychloroquine added to standard of care can shorten hospitalization and reduce the risk of non-invasive ventilation, admittance to ICU, and death.

## Study methods

### Trial design

The patients are enrolled in the trial only after obtaining informed consent. The trial is conducted at eight centers in Denmark:

1. Section of Respiratory Medicine, Department of Medicine, Herlev-Gentofte Hospital, University of Copenhagen, Herlev and Hellerup, Denmark; principal investigator (PI): Jens-Ulrik Jensen, MD PhD
2. Department of Respiratory Medicine, Hvidovre Hospital, University of Copenhagen, Hvidovre, Denmark; PI: Charlotte Suppli Ulrik Professor, MD DMSc.
3. Department of Pulmonary and Infectious Diseases, Hillerød Hospital, University of Copenhagen, Hillerød, Denmark; PI: Andrea Browatzki, MD
4. Department of Respiratory Medicine, Bispebjerg Hospital, University of Copenhagen, Copenhagen, Denmark; PI: Therese Lapperre, MD PhD
5. Department of Respiratory Medicine, Zealand Hospital, Næstved Hospital, University of Copenhagen, Næstved, Denmark; PI: Uffe Bødtger, MD PhD
6. Department of Respiratory Medicine, Odense University Hospital, University of Southern Denmark, Odense, Denmark; PI: Christian B. Laursen, MD PhD
7. Respiratory Medicine, Aarhus and Aalborg University; PI: Ulla Weinreich, MD PhD
8. Department of Respiratory Medicine, Zeeland University Hospital, Roskilde, PI: Christian Niels Meyer, MD PhD

Patients will be randomized to one of two treatment arms:

i) *Intervention group*: Days 1–3: azithromycin 500 mg × 1 od plus hydroxychloroquine 200 mg × 2 od; days 4–15: azithromycin 250 mg × 1 od plus hydroxychloroquine 200 mg × 2 od
ii) *Control group*: The control group will receive placebo for both types of intervention medications. If the investigational therapy (or part of) becomes standard treatment during the study, this may also be offered to the control group

The analyses described in this document will be performed by coordinating investigator Pradeesh Sivapalan, MD PhD (Section of Respiratory Medicine, Department of Medicine, Herlev-Gentofte Hospital, University of Copenhagen, Herlev and Gentofte, Denmark) in cooperation with the sponsor and principal investigator Jens Ulrik Jensen, once the data have been entered, cleaned, and released for use.

**Fig. 1:**
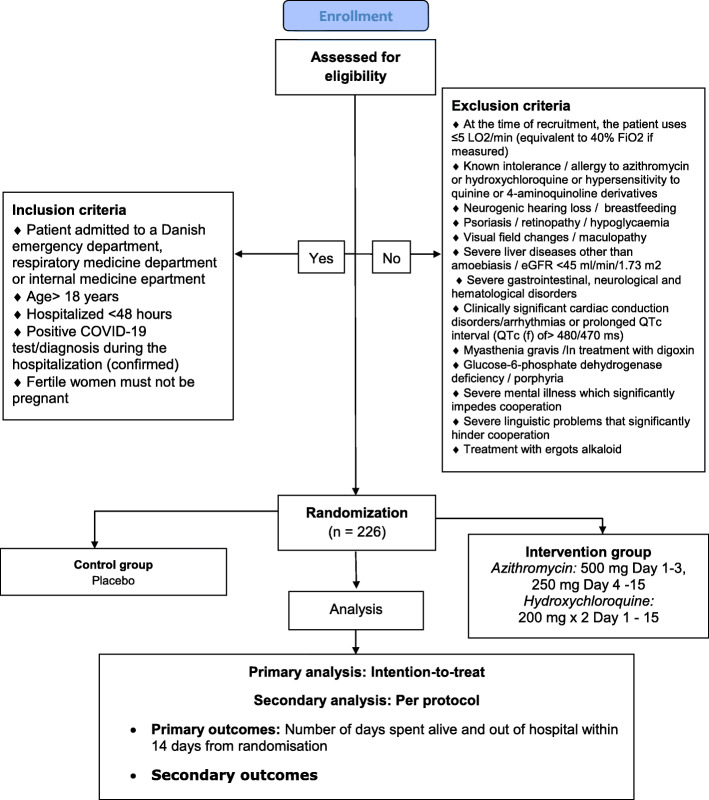
CONSORT transparent reporting of trials

This document provides a detailed description of the statistical analyses that will be performed for the evaluation of the primary and secondary endpoints of protocolized for the ProPAC-COVID study [6]. The analyses described in this document are compatible with the recommendations of the CONSORT 2010 statement [7] (Fig. 1). The International Conference on Harmonisation (ICH) of Good Clinical Practice (GCP) [1] and leading experts recommend that randomized clinical trials should be analyzed according to predefined outcomes and a predefined statistical analysis plan [2]. To prevent selective reporting of outcomes and data-driven analysis and to increase transparency, this paper describing the detailed statistical analysis plan for the ProPAC-COVID trial will be published while enrolment of patients and collection of data is still ongoing and before the database is accessed for trial results.

## Statistical principles

### Randomization

Randomization will be in blocks of unknown size and the final allocation will be stratified for age (> 70 vs. ≤ 70 years), site of recruitment, and whether the patient has any of the following chronic lung diseases (yes vs. no): chronic obstructive pulmonary disease, asthma, bronchiectasis, and interstitial lung disease.

### Analysis population

Data will be analyzed using intention-to-treat (ITT) principles and main analyses will also be subject to modified ITT analysis (started but not completed the study) and per protocol analysis (completed all interventions). When applying the ITT principle, all randomized patients will be analyzed in the groups to which they were originally allocated, regardless of whether they received the intended treatment or whether a protocol violation or protocol deviation occurred. Patients who withdraw consent for the use of their data will not be included in any analysis; only the study group originally randomized to and withdrawal of consent will be reported. A secondary analysis of the primary efficacy outcome will use a per protocol (PP) population. A CONSORT diagram of participants will be presented in the study.

### Sample size

The power to avoid a type II error is 80% (1-β) at a two-sided 5% significance level, using a *t* test for the primary outcome, and a group-sequential design allowing for one interim analysis at half target recruitment. This led to a sample size of 226 subjects. All confidence intervals (CIs) reported will be 95% CIs. Full details of the sample size calculation are described in the protocol article.

### Analysis software

All analyses will be performed using SAS software version 9.4.

### Statistical interim analysis and stopping guidance

Planned analyses of safety and efficacy data (interim analysis) will be evaluated when 113 patients have completed the study (completed 1-month follow-up). These assessments will be made by an independent Data and Safety Monitoring Board (DSMB). The interim analysis will focus on reporting the following: selected baseline data (those readily available from the baseline data list below), primary outcome (in an O’ Brien-Fleming Plot), and all-cause mortality at 30 days (chi-square or Fisher’s exact test, whichever appropriate). To adjust the type 1 error rate for multiplicity, we will use the O’Brien-Fleming method in a group-sequential design, resulting in an alpha level for the interim analysis at 0.0054 and for the final analysis at 0.0492. The DSMB will review the protocol and monitoring guideline, evaluate the attempts to recruit participants and participants’ risk, and, on the basis of interim analyses, make recommendations to investigators as to whether to continue the study. In addition, the DSMB may at any time require an extraordinary interim analysis.

## Trial population

### Descriptive analyses—baseline characteristics at study enrolment (defined as day 1)

The following baseline characteristics of the study population will be summarized separately within each randomized group:

- Age, median (interquartile ranges (IQR)), years
- Male sex, *n* (%)
- Ethnicity (Caucasian, African (incl. African-American), Asian, Inuit, unknown/other)
- Body mass index (kg/m^2^, median, IQR)
- Current smoker, *n* (%)
- Ex-smoker, *n* (%)
- Non-smoker, *n* (%)
- Pack-years history (median, IQR, years)
- Use of oxygen therapy, *n* (%)
- Use of continuous positive airway pressure, *n* (%)
- Use of non-invasive mechanical ventilation (NIV), *n* (%)
- Infiltrates on chest X-ray, *n* (%)
- Oxygen consumption: L/min (median, IQR)
- Oxygen consumption: fraction of inspired oxygen (FiO_2_ (median, IQR))

Clinical findings (daily measurements)

- Systolic blood pressure (mm Hg)
- Diastolic blood pressure (mm Hg)
- Heart rate, beats/min
- Oxygen saturation with high flow nasal cannula, median, IQR
- Respiratory rate, breaths/min
- Temperature (°C)

Biochemistry findings (daily measurements)

- Leukocyte count, × 10^9^ cells/L
- Blood eosinophil count, × 10^9^ cells/L
- Lymphocyte count, × 10^9^ cells/L
- CRP (mg/L)
- Fibrin D-dimer (ng/mL)
- Ferritin (μg/L)
- Lactate dehydrogenase (U/L)

Arterial blood gas (mean ± standard deviation), day 1 and day 4

- PCO_2_, mmHg
- PO_2_, mmHg
- HCO_3_^−^
- pH

Other lab findings

- QTc (F) (via electronic measurement on electrocardiogram (ECG) at baseline)

Comorbid conditions, *n* (%)

- Asthma
- COPD
- Bronchiectasis
- Interstitial lung disease
- Allergy
- Diabetes mellitus
- Previous myocardial infarction
- Heart failure
- Atrial fibrillation
- Chronic renal failure
- Essential hypertension
- Osteoporosis
- Peripheral vascular disease
- Cerebrovascular disease
- Hematological diseases
- Depression
- Past or present lung cancer
- Previous or present non-lung cancer
- Previous thrombo-embolic disease (deep vein thrombosis/pulmonary embolism)
- Liver failure
- Rheumatic diseases

### Follow-up data and handling of missing data

The percentage of patients followed for each outcome data parameter will be reported for all predefined outcomes (primary and all secondary). Exploratory outcome analyses will be planned by the study group on suggestion form the reviewers/editors etc. Percentage with followed/missing data will also be reported for these outcomes.

### Adherence data

*N* + % patients in both arms who:

- Started azithromycin
- Started hydroxychloroquine
- Completed azithromycin (all days)
- Completed hydroxychloroquine (all days)
- Completed both drugs (all days)

### Medication during hospitalization

- Type of antibiotics (non-study drugs)
  - Ciprofloxacin, piperacillin/tazobactam, ceftazidime, meropenem, colistin, gentamycin, amoxicillin, amoxicillin/clavulanic acid, roxithromycin, dicloxacillin, penicillin, azithromycin, or others
- Days with antibiotics—any (median days on any type)
- Days with corticosteroids (median days)

For continuous variables, means and standard deviations will be presented, when normally distributed, otherwise as medians and IQR. For categorical variables, the number and percentage of participants within each category will be presented. For each variable, the percent of missing values will be reported. For categorical values, chi-square and Fisher’s exact test will be used. For time-to-event variables, Cox regression and log-rank test will be used, and for the latter, a corresponding Kaplan-Meier plot will be presented.

## Data analysis

### Primary objective and outcome

The primary outcome is a continuous outcome: “days alive and out of hospital within 14 days (DAOH14) after recruitment.” The outcomes are defined as number of days spent alive and out of hospital within 14 days from randomization. This is sensitive outcome used in previous publications [8, 9]. Among other advantages, lead-time bias due to death was avoided using this endpoint measure (i.e., patients who died early would not be counted as a short length of stay). Data for the primary outcome will be analyzed using a general linear model adjusted for the stratification covariates (age, site, and chronic lung disease) and estimated means and difference in means with 95% CI will be presented. Additionally, for sensitivity analysis median with IQR with corresponding non-parametric test, e.g., Mann-Whitney *U* test will be presented. In the case of missing covariate data, multiple imputations will be used. The estimation from the study group is that DAOH14 will be a number above or equal to 4. If DAOH14 is < 4, DAOH at 21 days will be presented instead. Apart from the main analysis, the primary outcome analysis will be performed as an adjusted analysis using general linear models while adjusting for the following variables: age (per year increase), sex (M/F), COPD GOLD C/D (yes vs. no), heart failure NYHA III-IV (yes vs. no), and current smoker (yes vs. no).

### Secondary objective and outcomes

1. Categorization of hospitalization status [time frame: 14 days]

The patient will be categorized into one of the following 8 categories depending on status of their hospitalization. Only one category can be “yes”:

- Dead (yes/no)
- Hospitalized and receiving mechanical ventilation or extra corporal membrane oxygenation (yes/no)
- Hospitalized and receiving NIV or using high-flow oxygen device (yes/no)
- Hospitalized and given oxygen supplements different from (b) and (c) (yes/no)
- Hospitalized and without oxygen treatment, but receiving other treatment (both related to COVID-19 or other) (yes/no)
- Hospitalized for observation (yes/no)
- Discharged from hospital with restriction of activity level (yes/no)
- Discharged from hospital without any restrictions of activity level (yes/no)

For this analysis, the patient will be assigned a number between 1 and 8. Frequencies for the categories will be presented. A proportional odds logistic regression model will be applied. The key parameter of interest is the “common odds ratio,” which quantifies the shift in the severity distribution resulting from treatment. For an efficacious treatment, an odds ratio greater than 1 quantifies an improvement in disease severity; a value of 2 indicates a bigger improvement than a value of 1.25. If the preconditions for the proportional odds model are not considered fulfilled, we will present the frequencies and calculate *p* values for shifts in levels by a Wilcoxon rank-sum test.

2. Admitted to ICU, if admitted to ICU then length of stay [time frame: 14 days]

Number of patients admitted to intensive care will be compared using chi-square test. Length of stay in ICU will be analyzed using a *t* test. Days not alive within the time frame will be added to days at ICU. If days not alive are equal in the two treatment groups, we will further present days at ICU excluding days not alive.

3. Have required NIV during hospitalization [time frame: 14 days]

Use of NIV will be compared by a chi-square test.

4. Mortality [time frame: 30 days]

Differences in mortality will be calculated using Cox proportional hazards adjusting for the following variables: age (per year increase), sex (M/F), COPD GOLD C/D (yes vs. no), heart failure NYHA III-IV (yes vs. no), and active smoker (yes vs. no). Treatment effects will be presented as hazard ratios (HR) and 95% CIs. Furthermore, Kaplan-Meier plot will be presented in combination with the log-rank test. Patients will be censored in the case of lost to follow-up.

5. Length of hospitalization [time frame: 14 days]

Length of hospitalization between groups will be compared using a *t* test.

6. Days alive and discharged from hospital [time frame: 30 days]

This is equal to the primary endpoint but with a longer time frame and will be analyzed similarly.

7. Mortality [time frame: 90 days]—to be reported in a secondary publication

Differences in mortality will be calculated using Cox proportional hazards adjusting for the following variables: age (per year increase), sex (M/F), COPD GOLD C/D (yes vs. no), heart failure NYHA III-IV (yes vs. no), and active smoker (yes vs. no). Treatment effects will be presented as hazard ratios (HRs) and 95% CIs. Furthermore, Kaplan-Meier plot will be presented in combination with the log-rank test. Patients will be censored in the case of lost to follow-up. However, this is not expected, since follow-up for these values is 100.0% in the Danish health registry.

8. Mortality [time frame: 365 days]—to be reported in a secondary publication

Differences in mortality will be estimated using both unadjusted and adjusted analyses. For the adjusted analysis, a Cox proportional hazards model will be used and adjustment for the following variables will be done: age (per year increase), sex (M/F), COPD GOLD C/D (yes vs. no), heart failure NYHA III-IV (yes vs. no), and active smoker (yes vs. no). Treatment effects will be presented as HRs and 95% CIs. Furthermore, Kaplan-Meier plot will be presented in combination with the log-rank test. Patients will be censored in the case of lost to follow-up.

9. Time to readmission (all causes) [time frame: 30 days]
10. Differences in readmission will be calculated using Cox proportional hazards adjusting for the following variables: age (per year increase), sex (M/F), COPD GOLD C/D (yes vs. no), heart failure NYHA III-IV (yes vs. no), and active smoker (yes vs. no). Treatment effects will be presented as HRs and 95% CIs. Furthermore, Kaplan-Meier plot will be presented in combination with the log-rank test. Patients will be censored in the case of lost to follow-up. Number of days requiring NIV [time frame: 14 days]
11. Number of days on NIV will be compared using a *t* test. If patients die within 14 days, days of death will be counted as days in NIV. Change in patient’s oxygen partial pressure [time frame: 4 days]

Delta PaO_2_ measured in arterial blood gas analysis.

Changes will be calculated by an analysis of covariance (ANCOVA) method adjusting for baseline values.

12. Change in patient’s PCO_2_ partial pressure [time frame: 4 days]

Delta PaCO_2_ measured in arterial blood gas analysis.

Changes will be calculated by an ANCOVA method adjusting for baseline values.

13. Level of pH in blood [time frame: 4 days]

pH measured in arterial blood gas analysis.

Levels in pH will be compared using a *t* test.

14. Time to no oxygen supplement (or regular oxygen supplement “long-term oxygen therapy”) [time frame: 14 days]

Time to no oxygen supplement will be presented by the Kaplan-Meier method and differences calculated by log-rank test.

For all analyses using parametric statistics (*t* test, ANCOVA), the distribution will be inspected. Biochemical markers will be transformed if necessary whereas length of stays will not be transformed. If parametric statistics is considered inappropriate, a non-parametric alternative will be used. For analyses with a dichotomous outcome, Fisher’s exact test will be used if the chi-square test is not considered appropriate.

Arrhythmias:

- ECG: QTc: *n* (%) patients in both arms who at any time point after baseline had a QTc (F) > 500 ms. We will do separate analysis for men and women.
- *N* (%) ventricular arrhythmias (apart from ventricular extrasystoles and non-sustained ventricular tachycardia)

### Subgroup analyses (all according to baseline values)

Scheduled to perform the following stratified analyses for the primary outcome:

- Stratified analyzes in the presence of chronic lung disease or not
- Stratified analyzes for QTc across the median
- Stratified analyzes for < 2 L/min nasal oxygen vs. ≥ 2 L nasal oxygen
- Stratified analyzes CRP < 50 mg/L and CRP ≥ 50 mg/L
- Stratified analyzes D-dimer > 0.8 mg/L or D-dimer ≥ 0.8 mg/L
- Stratified for remdesivir treatment (yes or no)

### Figures and tables

The first figure will be a consolidated standards of reporting of randomized trials (CONSORT) flow chart. The second figure will be a Kaplan-Meier plot to describe the rate of death by treatment arms. The third figure will be a forest plot illustrating all the preplanned sub analyses. The first table will be the baseline characteristics of the ITT population (Table 1). The second table will include the primary and secondary outcomes according to the two allocation and pairwise comparisons.

**Table 1:**
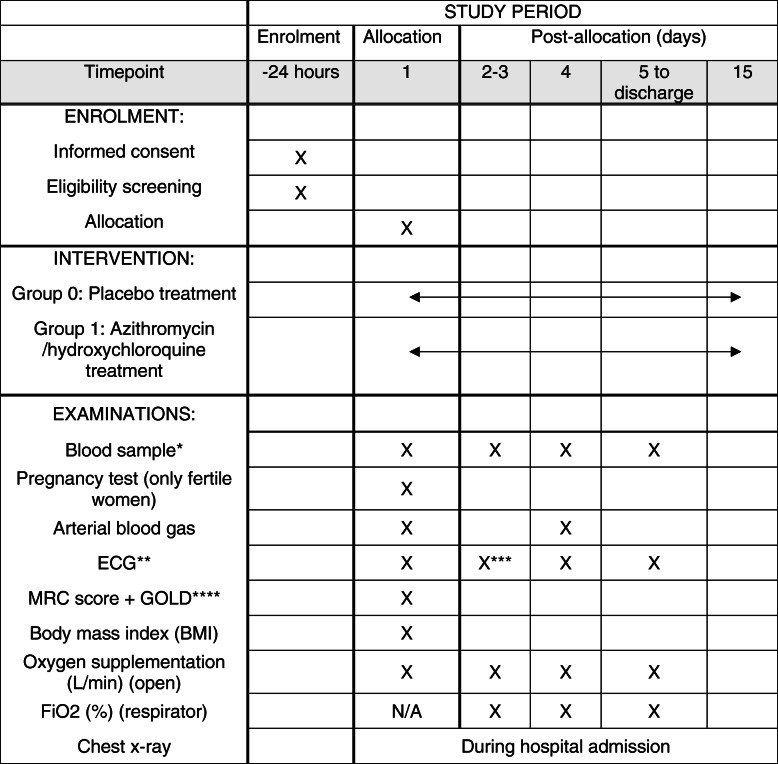
SPIRIT figure: overview of examinations that each participant will undergo

*The blood samples include hemoglobin (Hb), leukocytes + differential count, thrombocytes, C-reactive protein (CRP), Na+, K+, albumin, creatinine, urea, amylase, alkalic phosphatase, beta-2-microglubulin, fibrinogen, glucose, TSH, INR, bilirubin, D-dimer, APTT, calcium, triglycerides, ferritin, and lactate dehydrogenase (LDH). These blood tests will also be recommended daily for COVID patients outside studies in the recommendation of the Danish lung medicine association

**When screening for the study, any ECG from within the last 3 days can be used

***A follow-up ECG can be recorded during any remaining days of the hospital admission

****Only in patients with COPD

### Blinding of the statistician

The detailed analysis plan was written in strict concordance with the trial protocol approved by the regulatory authorities prior to recruitment initiation. The entire statistical analysis plan was published at www.coptrin.dk before the trial was finalized (before the database was closed). All analyses will be done prior to breaking of the randomization code (analysis comparisons between “arm A” and “arm B” (random names). The coordinating investigator (PS) and the study sponsor and principal investigator (JUJ) will conjointly perform all the data analyses according to this plan, except the interim analyses which will be performed by Dr. Josefin Eklöf (who is not an investigator of this trial) from Section of Respiratory Medicine, Department of Medicine, Gentofte Hospital, University of Copenhagen, Hellerup, Denmark. An unblinding date will be chosen and published online at www.coptrin.dk, and on this date, the allocation will be unblinded. After unblinding of the allocation, further analysis will not be done, except on request from reviewers/editors handling submitted papers.

## Data Availability

Information regarding subjects is processed and stored in accordance with the Data Protection Regulation (GDPR), the Data Protection Act, and the Health Act, and the project is properly notified in accordance with applicable rules and laws to the appropriate authorities.

## Abbreviations

Cis: Confidence intervals
COPD: Chronic obstructive pulmonary disease
ANCOVA: Analysis of covariance
CONSORT: Consolidated standards of reporting of randomized trials
DAOH14: Days alive and out of hospital within 14 days after recruitment
GCP: Good Clinical Practice
HRs: Hazard ratios
ICH: International Conference on Harmonisation
ITT: Intention-to-treat
IQR: Interquartile range
NIV: Non-invasive ventilation
